# Harnessing halophyte phytochemicals and biostimulants to enhance crop resilience under climate stress: a comprehensive review

**DOI:** 10.1007/s11356-026-38075-2

**Published:** 2026-08-27

**Authors:** Muziri Mugwanya, Fahad Kimera, Mahmoud Zeid, Hani Sewilam

**Affiliations:** 1 Center for Applied Research On the Environment and Sustainability (CARES), School of Science and Engineering, The American University in Cairo, AUC Avenue, P.O. Box 74, New Cairo, 11835 Egypt; 2 Crop Science Department, Faculty of Agriculture-Shatby, Alexandria University, Alexandria, Egypt; 3 UNESCO Chair in Hydrological Changes and Water Resources Management, Department of Engineering Hydrology, RWTH Aachen University, Aachen, Germany

**Keywords:** Saline agriculture, Secondary metabolites, Rhizosphere engineering, Biogenic pesticides, Osmotic adjustment, Halophyte biorefinery

## Abstract

Global food security is increasingly compromised by the synergistic pressures of soil salinization and the climate-mediated proliferation of resilient agricultural pests and weeds. Halophytes, extremophile plants naturally adapted to high-salinity niches, represent an underexploited reservoir of bioactive compounds and microbial consortia with significant bioeconomic potential. This review synthesizes the multi-functional utility of halophyte-derived bioactive compounds in mitigating contemporary environmental stressors through two primary lenses. First, we discuss the biochemical efficacy of halophyte-derived secondary metabolites and essential oils (EOs) as sustainable biopesticides, highlighting their potency against taxa that exhibit increasing resistance to conventional synthetic inputs. Second, we evaluate the mechanistic role of halophyte-associated rhizobiomes and biostimulants in enhancing the physiological plasticity of glycophytic crops. Specifically, we detail their capacity to modulate antioxidant defense systems, maintain ion homeostasis, and alleviate osmotic stress under saline environments. Lastly, we delineate the phytochemical stability and non-target safety of halophyte plant extracts and essential oils. By synthesizing current ecotoxicological data, we illustrate how these biogenic inputs offer superior selectivity, safeguarding beneficial entomofauna and soil microbiota while maintaining high efficacy against target insect pests. By bridging the traditionally disparate domains of phytochemical pest control and rhizosphere engineering, this review proposes a holistic biotechnological paradigm. We conclude that the transition toward halophyte-based solutions offers a strategic, circular-economy pathway to stabilize food systems and safeguard public health, providing a robust bio-based alternative to synthetic agrochemicals in increasingly marginalized landscapes.

## Introduction

For more than 8 decades, disruptions in the global carbon cycle have been extensively studied and quantified with current records indicating an increase in atmospheric carbon dioxide (CO_2_) concentrations reaching ~ 420 parts per million (ppm) (Neubauer 2022). The increase in atmospheric CO_2_ concentrations is mainly attributed to human activities such as the burning of fossil fuels and changes in land-use management (Zhou et al. 2019; Zhao et al. 2021). Nonetheless, the increase in CO_2_ concentrations in the atmosphere is regarded as the major contributor to climate change whose consequences are as follows: increasing ocean and atmospheric temperatures, melting of glaciers, rise in the sea level, ocean acidification, and changes in the intensity and frequency of precipitation (Gao et al. 2019; Lee et al. 2021). All these changes have an impact on the Earth’s ecosystems whose disrupted structure, stability, and function have a direct effect on people’s well-being and livelihoods across the globe (Neubauer 2022). For example, the increasing atmospheric temperature (i.e., global warming) and changes in the intensity and frequency of precipitation have been associated with the emergence of exotic disease vectors in non-endemic regions (Conn et al. 2002; Khasnis and Nettleman 2005) and insect pests (Saidana et al. 2007; Qureshi et al. 2018). This has negatively impacted human health and activity such as agriculture due to enhanced insect survival and resistance to synthetic insecticides (Soummane et al. 2011; Wang et al. 2020; Basile et al. 2022). Likewise, global warming, coupled with increasing CO_2_ concentrations in the atmosphere, has also been linked to genetic modifications in plants, particularly weeds, which have led to their increased survival, spread, and resistance to synthetic herbicides (Clements and Ditommaso 2011; Mou et al. 2025). Weeds compete with plants of economic importance for water and nutrients thus leading to a reduction in crop yields (Clements and Ditommaso 2011). Crop yields have also been affected by long droughts and increasing soil salinity as a result of reduced precipitation, high temperatures, intrusion of seawater on land, and/or irrigation of crops with saline water leading to severe land degradation (Clements and Ditommaso 2011).

Domestication of halophyte plants could aid in reducing plant disease incidence and the spread of weeds, owing to the inherent genetic makeup that enables them to tolerate harsh environmental conditions. They possess several ecological, anatomical, morphological, physiological, and biochemical traits that allow them to survive in highly saline (i.e., ≥ 200 mM NaCl) and heavy metal contaminated environments, high temperatures, drought, and elevated CO_2_ concentrations in the atmosphere (Hasanuzzaman et al. 2014; Jothiramshekar et al. 2018). This review, therefore, aims to discuss (1) the utilization of halophyte plant extracts and essential oils (EOs) in controlling the spread of insect pests and weeds that have become a menace to man and food production under the current climate change scenarios, (2) the application of halophyte plant biostimulants and root-associated microbiome aimed at ameliorating salinity stress effects in sensitive crops, and (3) environmental persistence and ecotoxicological impacts on non-target organisms. Our review will therefore give new insight into the economic importance of halophyte plants in the era of climate change.

## Plant resilience and crop protection

In the search for sustainable alternatives to synthetic agrochemicals, halophyte-derived extracts and essential oils have emerged as potent agents for biogenic crop protection (Agrawal et al. 2025). These extremophile plants synthesize a sophisticated array of secondary metabolites, including phenolic acids, flavonoids, and volatile terpenoids, as a defensive response to the oxidative stress inherent in saline habitats (Machado et al. 2024). When repurposed for agriculture, these bioactive fractions exhibit multi-faceted efficacy, functioning as natural pesticides, including insecticidal and herbicidal activities. Unlike conventional pesticides that often rely on a single active ingredient, the complex chemical profiles of halophyte oils provide a synergistic “cocktail” effect, which significantly hinders the development of resistance in target pests (Pavela et al. 2020). By leveraging these bio-based resources, we can transition toward a circular agricultural model that safeguards biodiversity while maintaining high yields in increasingly degraded environments.

### Control of insect pests

Several environmental factors such as temperature, humidity, precipitation, and CO_2_ levels influence the insects’ life cycle, population dynamics, and geographical distribution (Skendžić et al. 2021; John et al. 2024; Nega 2025). Any variation in environmental temperatures, for instance, can affect the insect’s feeding habits (i.e., accelerated feeding), fecundity, generation time, and survival (Skendžić et al. 2021). In comparison to soil-dwelling species, insects that spend most of their life above the soil surface are more susceptible to temperature fluctuations (Skendžić et al. 2021). As such, current global warming scenarios have indicated an increase and decrease in insect pest infestations of certain species in temperate and tropical regions, respectively (Deutsch et al. 2008; Shrestha 2019). On the other hand, the survival and distribution of soil-dwelling insect pests are dependent on the intensity and amount of precipitation (Thanuja et al. 2025). Seasonal floods as a consequence of climate change often lead to the displacement of insect eggs and larvae to new areas thus leading to the emergence of insect pest infestations and exotic disease outbreaks (Skendžić et al. 2021).

**Fig. 1:**
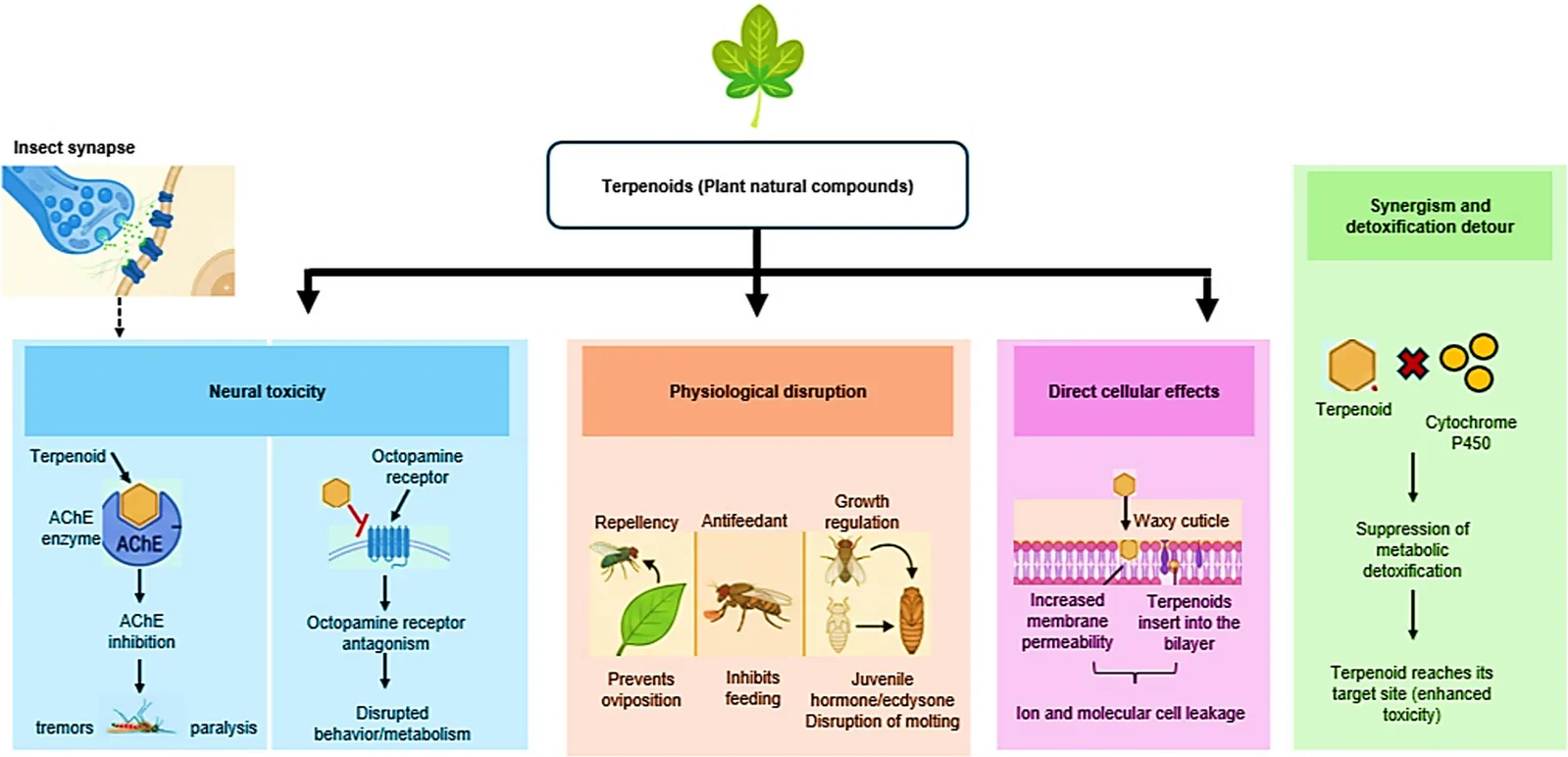
Conceptual framework of the multi-target insecticidal mechanisms of terpenoids in insect pest control. The bioactivity of these compounds is characterized by systemic physiological failure. Neurotoxicity is achieved through the inhibition of acetylcholinesterase (AChE), which prevents the degradation of acetylcholine; the resulting synaptic accumulation triggers continuous muscular over-excitation, paralysis, and mortality. Furthermore, terpenoids may act as ligands for octopamine receptors, disrupting critical behavioral and metabolic pathways. Endocrine disruption occurs via structural mimicry of essential growth hormones: sesquiterpenoids functioning as juvenile hormone (JH) analogs can arrest pupation or induce the formation of non-viable larval-pupal intermediates, while JH antagonists and triterpenoid ecdysone agonists may result in premature, lethal molting cycles. At the cellular level, the lipophilic nature of terpenoids allows for integration into lipid bilayers, compromising membrane integrity and leading to the efflux of essential ions and loss of cellular homeostasis. Critically, many terpenoids enhance their own efficacy by inhibiting cytochrome P450 monooxygenases, thereby suppressing metabolic detoxification and ensuring the active constituents reach their primary molecular targets

With increasing resistance to synthetic insecticides, controlling insect pests has been a challenge in the agricultural sector. Moreover, the increasing environmental temperatures have been shown to accelerate the photodegradation and volatilization processes of pesticides, thus leading to the application of higher doses as a strategy for climate change adaptation (Delcour et al. 2015; Bustos et al. 2019; Rhodes and McCarl 2020; Zinyemba et al. 2021). However, there have been reports of increasing toxicity and pest resistance to synthetic pesticides, and hence, more environmentally friendly alternatives than synthetic pesticides are warranted (Maino et al. 2018; Kneeshaw et al. 2021; Ma et al. 2021). The use of halophyte plant extracts and EOs in the control of insect pests is recently gaining attention with more studies focused on controlling insect pests that attack stored grains. A previous study by Wang et al. (2020) showed that exposure of *Tribolium castaneum*, *Callosobruchus maculatus*, and *Sitophilus oryzae* to EOs obtained from aerial parts of *Lobularia maritima* resulted in high mortalities of adults of *C. maculatus* (LC_50_ = 7.48 µL L^−1^) with a moderate insecticidal effect on *T. castaneum* (LC_50_ = 59.94 µL L^−1^) and *S. oryzae* (LC_50_ = 35.37 µL L^−1^). GC-MS revealed the presence of azeleonitrile (39.7%), *trans*-*3*-pentenenitrile (36.3%), and 4-isothiocyanato-1-butene (10.9%) as the most abundant compounds in the EO of *Lobularia maritima.* The authors found strong fumigant effects of trans-3-pentenenitrile on all the tested insect species. *trans*−3-pentenenitrile functions as a strong respiratory toxin, killing insects through disruption of cellular respiration. Saidana et al. (2007) evaluated the insecticidal activity of different salt marsh plant extracts of *Tamarix boveana*,* Frankenia laevis*,* Suaeda fructicosa*, and *Statice echioides* on larvae and adults of *Tribolium confusum*. Chloroform, ethyl acetate extracts of *F. laevis*, *S. echioides*, and *T. boveana*, and petroleum ether extracts of *F. laevis* showed significant inhibition in larval growth and strong antifeedant properties. In another study, Qureshi et al. (2018) obtained root and leaf extracts of *Salvadora persica* L. and tested their pest control potential against the 6th instar larvae and adult stages of *T. confusum*. The authors observed a 50% mortality rate in root extracts against the larvae and adults at concentrations of 0.014 mL g^−1^ and 0.16 mL g^−1^, respectively. For leaf extracts, a 50% mortality rate against larvae and adults was observed at concentrations of 0.012 mL g^−1^, 0.12 mL g^−1^, and 0.16 mL g^−1^, respectively. Acheuk et al. (2018) investigated the insecticidal and repellent activity of crude ethanolic extracts of *Halocnemum strobilaceum* against adults of red flour beetle *Tribolium castaneum*. The authors observed the highest repellent activity and 100% mortality in *T. castaneum* adults at a concentration of 1000 µg insect^−1^. Likewise, inhibition of acetylcholinesterase (AChE) activity was observed at 1000 µg insect^−1^. Phytochemical screening of the plant extract revealed the presence of alkaloids, antocyans, gallic tannins, flavonoids, saponins, and coumarins. A recent study by Basile et al. (2022) has indicated that exposing *S. oryzae* and *Rhyzopertha dominica* adults to EOs obtained from *Calendula incana *subsp.* maritima* resulted in high mortalities of 65.5% and 44%, respectively. GC × GC-MS analysis of *Calendula incana *subsp.* maritima* EOs revealed an abundant presence of cubebol (35.39%), 4-epi-cubebol (22.99%), and cubenol (12.77%) which the authors anticipate could be responsible for the observed toxicity effects in the tested storage insect pests. As the most expansive and structurally diverse group of plant secondary metabolites, terpenoids function as a critical component of natural defense mechanisms against insect herbivory. In contrast to conventional synthetic insecticides, which typically operate on a single molecular target, terpenoids frequently employ a multifaceted, multi-target mode of action. This mechanistic complexity is thought to significantly hinder the selection for resistance within pest populations. The bioefficacy of a particular terpenoid is governed by its chemical structure, volatility, and the specific route of exposure (e.g., ingestion, topical contact, or inhalation) (Câmara et al. 2024). The generalized inhibitory pathways of terpenoids against insect pests are subdivided into four primary categories, as depicted in Fig. 1.

Studies on the use of halophyte plant extracts and EOs to control other economically important agricultural insect pests are scarce, and only a few studies have addressed this problem. For example, Suresh et al. (2020) investigated the effect of encapsulating sea fennel (*Crithmum maritimum*) EO in nanoemulsion for controlling the cotton leafworm (*Spodoptera litura*) and reported significant toxicity of sea fennel EO, nanoemulsion, and SiO_2_ nanoparticles against the larval and pupal stages of *S. litura*. Encapsulated SiO_2_ nanoparticles showed LC_50_ values ranging from 24.610 (1st instar) to 64.546 µL L^−1^ (pupae) on *S. litura*. It was also noted that all the tested forms of bioinsecticides reduced the fecundity and longevity of *S. litura*. Similarly, Pavela et al. (2020) demonstrated significant insecticidal activity of *Emex spinosa* essential oils against the African cotton leafworm, *Spodoptera littoralis*, reporting a median lethal dose (LD_50_) of 55.6 µg larva⁻^1^. Furthermore, the entomotoxic potential of halophytic species was evaluated by Soummane et al. (2011), who examined the effects of dichloromethane and methanol extracts from *Salicornia arabica*, *Suaeda fruticosa*, and *Tamarix gallica* on the Mediterranean fruit fly, *Ceratitis capitata*. Their findings revealed that both adults and early-instar larvae exhibited greater susceptibility to methanolic fractions than to dichloromethane extracts. Notably, the methanol extract of *T. gallica* displayed the most potent bioactivity, which was attributed to a high concentration of secondary metabolites, specifically alkaloids, tannins, and saponins, identified during phytochemical screening. Complementary results were observed by Kabaru and Gichia (2001) regarding the desert locust, *Schistocerca gregaria*. Their research indicated that exposure to extracts from *Rhizophora mucronata* induced high adult mortality, a phenomenon primarily driven by the extract’s potent antifeedant properties.

### Control of weeds

There is no doubt that fluctuations in weather events have a profound effect on plant physiology and genetics (Ramesh et al. 2017). The ever-changing environment, characterized by rising temperatures and atmospheric CO₂ concentrations, has imposed selective pressures that have shaped the phenotypic plasticity and evolutionary adaptations of plants, enhancing their capacity to tolerate multiple environmental stressors (Clements and Ditommaso 2011; Ramesh et al. 2017). Weeds easily and quickly build up a tolerance to stress compared to crops of economic interest and hence cases of weed invasions and crop-yield losses are expected to increase in the future (Ramesh et al. 2017; van Boheemen et al. 2018; Cheng et al. 2020). The application of synthetic herbicides is one of the most commonly used approaches in controlling weeds. However, recent reports have indicated increasing synthetic herbicide resistance and loss of herbicidal efficacy due to the changing environment (Ofosu et al. 2023; Mohod et al. 2025). Moreover, the production chain of synthetic herbicides, their transportation, and their field applications impact climate change. For instance, industrial production of herbicides leads to the release of greenhouse gases (GHGs) such as methane (CH_4_), nitrous oxide (N_2_O), and carbon dioxide (CO_2_), whose role in global warming has been documented (Cornish et al. 2024; Brookes 2026). Therefore, there is a need to search for climate-resilient and environmentally friendly alternatives to control weeds amid climate change.

**Fig. 2:**
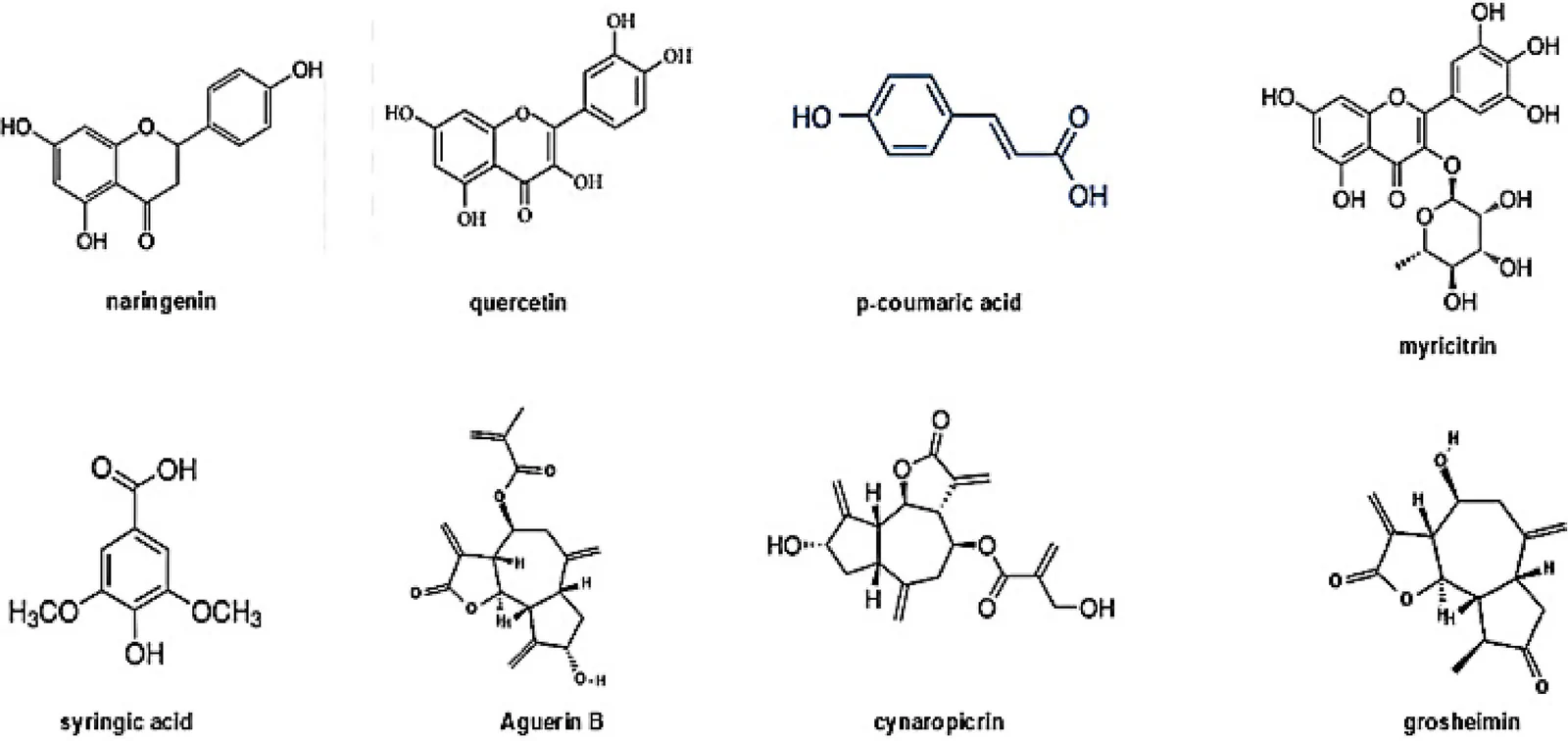
Chemical structure of bioactive compounds in methanolic and ethyl acetate extracts of *C. cardunculus*

**Fig. 3:**
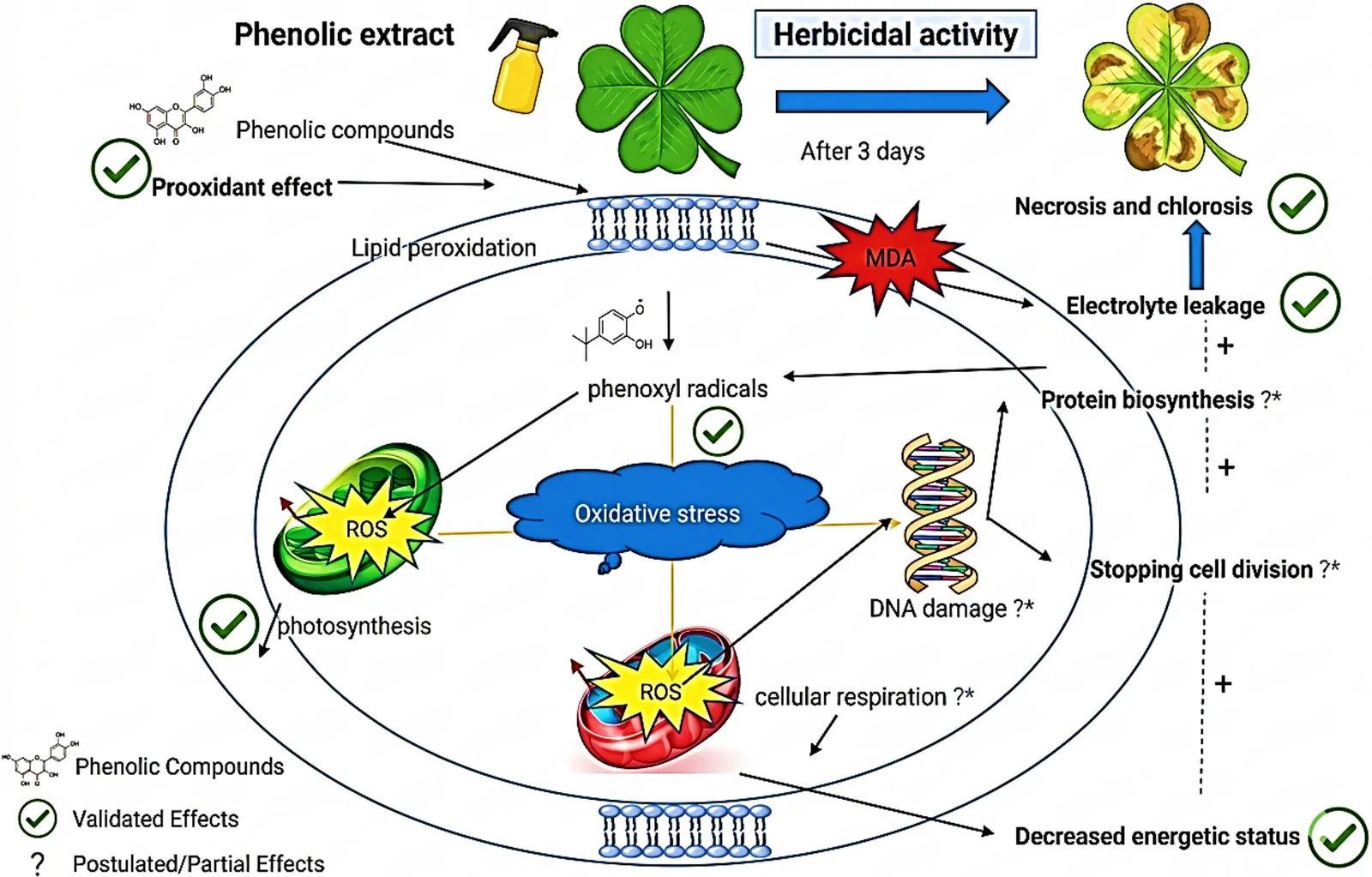
Schematic representation of phenolic-induced phytotoxicity. The association of phenolic compounds with the hydrophilic region of the membrane triggers the formation of phenoxyl radicals. This oxidative stress pathway impairs photosynthesis via the overproduction of ROS. Modified image obtained from Kaab et al. (2020a)

The exploration of plant secondary metabolites as sustainable alternatives to synthetic herbicides has emerged as a significant focal point in agricultural research. However, the application of halophyte-derived extracts for weed management remains in its nascent stages, with only a limited number of studies documenting their herbicidal efficacy. Research by Omezzine et al. (2011) highlighted the potent phytotoxicity of *Inula crithmoides* L. extracts. Their findings demonstrated that crude extracts from leaves and flowers (applied at 40 g L⁻^1^) induced complete mortality in peganum and 66.5% mortality in thistle. Furthermore, chloroform-based fractions exhibited peak toxicity against thistle seedlings at 6000 ppm, while the soil incorporation of leaf and flower powder (2.5 g kg⁻^1^) significantly suppressed the growth of both target species. Recent investigations have delved into the mechanisms of action underlying these effects. For instance, Kaab et al. (2020a) evaluated the facultative halophyte *Cynara cardunculus*, reporting that its crude extracts induced significant oxidative stress in treated plantlets. This physiological disruption was largely attributed to bioactive flavonoids, specifically myricitrin and naringenin. Subsequent research by Kaab et al. (2020b) confirmed the broad-spectrum efficacy of *C. cardunculus* methanolic extracts against *Phalaris minor*, *Silybum marianum*, and *Trifolium incarnatum*. Phytochemical profiling identified a suite of active phenolic compounds, including p-coumaric acid, syringic acid, and quercetin, as primary drivers of germination inhibition. The herbicidal profile of *C. cardunculus* was further refined by Rial et al. (2014), who tested ethyl acetate extracts against invasive species such as *Brachiaria* sp. and *Echinochloa crus-galli*. The study observed profound growth suppression, which was linked to specific sesquiterpene lactones isolated during fractionation (e.g., aguerin B, cynaropicrin, and grosheimin). Collectively, these studies underscore the untapped potential of halophytic secondary metabolites as bio-herbicidal agents, though further research is required to optimize their application and clarify their environmental persistence. Figure 2 shows bioactive compounds reported in methanolic and ethyl acetate extracts of *C. cardunculus*, and Fig. 3 illustrates a suggested mechanism for the herbicidal activity of phenolic substances.

The phytotoxic efficacy of the phenolic-rich extract is primarily driven by its role as a potent pro-oxidant within target plant tissues. As illustrated in Fig. 3, the hypothetical mechanism of herbicidal action involves the rapid induction of systemic, uncontrolled oxidative stress. This biochemical disruption originates within the chloroplasts and mitochondria, where it triggers an acute accumulation of reactive oxygen species (ROS). A critical, validated consequence of this redox imbalance is the initiation of extensive lipid peroxidation, characterized by a marked elevation in malondialdehyde (MDA) concentrations. The subsequent degradation of lipid bilayers compromises membrane integrity, leading to significant electrolyte leakage. This loss of cellular compartmentalization directly correlates with the macroscopic onset of necrosis and chlorosis observed 72 h post-treatment. Furthermore, the extract significantly impairs the plant’s energetic status, resulting in a sharp decline in metabolic viability. While the primary oxidative damage is well-documented, the observed reduction in plant vigor may be compounded by secondary impacts on protein biosynthesis, mitotic activity, and genomic integrity. Although these latter pathways remain postulated and require further molecular characterization, the current data confirm that the herbicidal activity of the phenolic extract is achieved through the lethal disruption of cellular bioenergetics and membrane stability via catastrophic oxidative damage.

### Halophyte plant extracts and root-associated microbes as plant biostimulants under salinity stress conditions

Climate change has exacerbated global freshwater scarcity and soil salinization, presenting formidable challenges to agricultural productivity (Khondoker et al. 2023; Tarolli et al. 2024). Salinity stress impedes plant development through a multi-faceted mechanism involving osmotic stress, nutritional imbalances, and ion cytotoxicity. Specifically, the excessive accumulation of sodium (Na^+^) and chloride (Cl^−^) ions disrupts essential metabolic pathways, ultimately resulting in irreversible cellular damage (Alharbi et al. 2022; Balasubramaniam et al. 2023; Abdelkader et al. 2024). Furthermore, the increasing reliance on saline water for irrigation, necessitated by the depletion of freshwater resources, deteriorates soil structure and health, creating a feedback loop that further diminishes crop yields (Wang et al. 2023; Kakabouki et al. 2026).

In response to these abiotic constraints, the application of plant biostimulants (PBs) has emerged as a promising mitigation strategy. As defined by the European Biostimulant Industry Council (EBIC), PBs are substances or microorganisms applied to plants or the rhizosphere to catalyze natural processes that enhance nutrient acquisition, improve stress tolerance, and enhance crop quality (Calvo et al. 2014; Del Buono 2021). Botanically derived PBs, in particular, are rich in diverse bioactive constituents, including minerals, vitamins, amino acids, poly- and oligosaccharides, and humic compounds, which collectively facilitate plant resilience and growth under adverse conditions (Gonçalves et al. 2026; Kutasy-Takács et al. 2026). Halophytes serve as a prolific reservoir of bioactive compounds, sparking significant interest in the application of their extracts to bolster crop productivity and resilience under environmental constraints. Recent research by Osman et al. (2021) explored the protective potential of *Arthrocnemum macrostachyum* extracts on soybean (*Glycine max* L.) under varying salinity regimes (0, 75, and 150 mM NaCl). The study demonstrated that foliar application significantly enhanced vegetative growth and survival rates. Furthermore, treated plants exhibited superior biochemical profiles, characterized by high concentrations of soluble sugars, soluble proteins, and photosynthetic pigments in both saline and non-saline conditions. The efficacy of halophytic extracts is often influenced by the method of delivery. Muniswami et al. (2023) evaluated the impact of seagrass extracts on okra (*Abelmoschus esculentus*), noting distinct morphological responses based on the application technique. Foliar spray applications primarily promoted reproductive growth, resulting in a higher frequency of flowers and pods, whereas soil drenching favorably influenced pod development, yielding significant increases in both average weight and length compared to foliar treatments. In addition to growth promotion, halophyte extracts play a critical role in modulating physiological stress responses. Sadasivam et al. (2017) found that liquid extracts from the seagrass *Zostera marina* enhanced salinity tolerance in tomato plants. This resilience was attributed to the upregulation of key enzymatic antioxidants, including superoxide dismutase (SOD), catalase (CAT), and ascorbate peroxidase (APX). These findings suggest that halophyte-derived biostimulants provide a robust defense mechanism against oxidative damage, thereby stabilizing plant performance in saline environments**.**

**Fig. 4:**
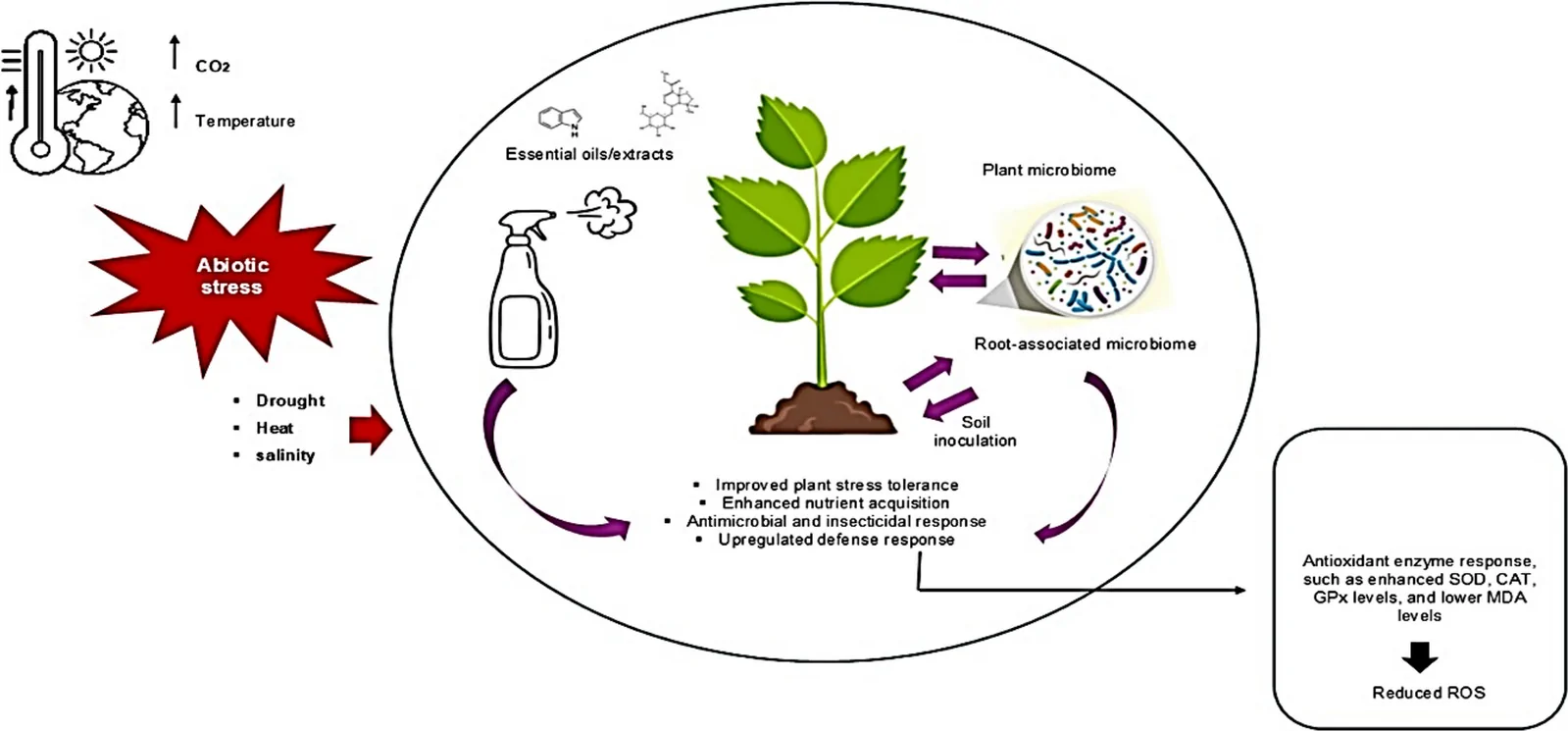
Conceptual figure summarizing plant-microbe-bioactive-stress interactions under climate change

Root-associated microorganisms play a critical role in mitigating the deleterious effects of abiotic stress on plant development (Muhammad et al. 2024; Solomon et al. 2024; Ullah et al. 2025). Leveraging rhizosphere interactions presents a sustainable, ecologically sound framework for food production (Tarroum et al. 2021), potentially reducing the agricultural sector’s reliance on synthetic fertilizers, which are major drivers of anthropogenic climate change (Mahmud et al. 2021; Zuluaga et al. 2024; Feng et al. 2026). The application of beneficial microbes improves soil structure and fertility while simultaneously enhancing crop yield and stress tolerance (Wei et al. 2024; Xing et al. 2025; Zhang et al. 2025). Figure 4 illustrates the conceptual framework of plant-microbe-bioactive-stress interactions under climate change. Halophyte rhizospheres are particularly rich in specialized microorganisms adapted to extreme conditions (Table 1). For example, inoculation of maize with *Providencia vermicola* BR68 and *Sarocladium kiliense* FS18 (isolated from *Suaeda salsa*) enhanced salinity resistance by increasing the activity of antioxidant and soil microbiome enzymes, such as catalase and sucrase (Wang et al. 2022). A bacterial consortium (*Bacillus zhangzhouensis* HPJ40 and *Pseudarthrobacter oxydans* SRT15) derived from *Salicornia ramosissima* and *Atriplex portulacoides* significantly promoted the growth of Swiss chard under saline stress (85 mmol L⁻^1^ NaCl), with rice and strawberries exhibiting the most pronounced positive responses (Redondo-Gómez et al. 2022).

**Table 1:** Some of the microorganisms isolated from the rhizosphere of halophyte plants

Halophyte Plant	Root-associated microorganisms	Reference
*Suaeda salsa*	*Providencia vermicola* BR68 and *Sarocladium kiliense* FS18	Wang et al. (2022)
*Salicornia ramosissima* and *Atriplex portulacoides* L	*Bacillus zhangzhouensis* HPJ40 and *Pseudarthrobacter oxydans* SRT15	Redondo-Gómez et al. (2022)
*Sporobolus montevidensis*	*Pseudomonas composti* SDT3, *Aeromonas aquariorum* SDT13, *Bacillus thuringiensis* SDT14	Redondo-Gómez et al. (2022)
*Allenrolfea occidentalis*	*Vibrio kanaloae* RA1, *Pseudoalteromonas prydzensis* RA15, *Staphylococcus warneri* RA18	Redondo-Gómez et al. (2022)
*Sporobolus maritimus*	*Bacillus methylotrophicus* SMT38, *Bacillus aryabhattai* SMT48, *Bacillus licheniformis* SMT51	Redondo-Gómez et al. (2022)
*Atriplex portulacoides*	*Vibrio spartinae* HPJ2, *Marinobacter sediminum* HPJ15, *Vibrio parahaemolyticus HPJ50*	Redondo-Gómez et al. (2022)
*Salicornia europaea*	*Vibrio neocaledonicus* SRT1, *Thalassospira australica* SRT8, and *Pseudarthrobacter oxydans* SRT15	Redondo-Gómez et al. (2022)
*Distichlis spicata*	*Pseudomonas lini* and *Bacillus* sp.	Palacio-Rodríguez et al. (2017)
*Salicornia bigelovii*	*Streptomyces chartreusis*, *Streptomyces tritolerans*, and *Streptomyces rochei*	Mathew et al. (2020)
*Limonium sinense*	*Streptomyces* sp. KLBMP5084	Gong et al. (2020)
*Aeluropus littoralis*	*Penicillium melinii* (A8) and *Byssochlamys spectabilis* (A5.1)	Tarroum et al. (2021)

The efficacy of these halophilic isolates is often linked to specific plant growth-promoting (PGP) traits. For example, strains of *Pseudomonas lini* and *Bacillus* sp. isolated from *Distichlis spicata* improved the growth of watermelon, *Arabidopsis*, and cucumber. This enhancement correlated with the microbial production of indole-3-acetic acid (IAA), siderophores, and increased phosphate solubilization (Palacio-Rodríguez et al. 2017). In *Salicornia bigelovii*, inoculation with a *Streptomyces* consortium increased seed yield by 79.7% and total biomass by over 60%, highlighting the potential for synergistic microbial interactions (Mathew et al. 2020). Similarly, *Streptomyces* sp. KLBMP5084 from *Limonium sinense* has been shown to bolster salinity tolerance in tomato seedlings (Gong et al. 2020).

Beyond bacterial inoculation, halophilic fungi and their metabolic byproducts offer innovative avenues for stress amelioration. Tarroum et al. (2021) observed that cell-free filtrates (CFFs) from *Penicillium melinii* and *Byssochlamys spectabilis* stimulated biomass production in hydroponically grown tobacco. The authors hypothesized that the presence of IAA within the CFFs served as the primary catalyst for this stimulatory effect, suggesting that purified microbial metabolites may provide a consistent alternative to live inoculants.

## Environmental persistence and ecotoxicological impacts on non-target organisms

While plant biopesticides (PBs) are frequently marketed as “green” alternatives to synthetic agrochemicals, recent evidence suggests that their “natural” origin does not inherently preclude non-target toxicity (Pereira et al. 2025; Soares et al. 2026). The primary environmental advantage of PBs, such as their rapid degradation under UV light and thermal instability, also presents a “persistence paradox” where acute high-concentration exposure can occur before metabolic breakdown (Pereira et al. 2025).

Although these characteristics reduce long-term environmental persistence, they may also limit the field efficacy of halophyte-derived bioactive compounds. Unlike controlled laboratory conditions, field environments expose PBs to UV radiation, rainfall, fluctuating temperatures, wind, and soil adsorption, all of which can accelerate degradation, reduce residual activity, and diminish pest control performance (Senthil-Nathan et al. 2026). Consequently, promising laboratory results may not always translate into consistent field efficacy, highlighting the need for optimized formulations, such as encapsulation and nano-delivery systems, together with well-designed multi-location field trials to validate performance under diverse agroecological conditions. These environmental factors should also be considered when evaluating ecological risks, as altered persistence and exposure patterns may influence interactions with non-target organisms.

The impact of PBs on pollinators, particularly *Apis mellifera* (honey bees), remains a focal point of recent ecotoxicological research (Catania et al. 2023). Terpenoids, a diverse class of natural compounds found in plants, can have varying effects on bees, ranging from beneficial to toxic, depending on the specific compound, concentration, and exposure method (Boncan et al. 2020). While many terpenoids serve as plant defenses, some are generally safe or only moderately harmful to bees, whereas others, particularly high-concentration monoterpenoids, can cause mortality (Sittichok et al. 2025). Menthol and thymol are considered more toxic to honey bees than other monoterpenoids (Caren et al. 2025). Recent studies on essential oil (EO) nanoformulations suggest that while these technologies improve stability, they may also increase the voracity or alter the foraging behavior of predatory mites, leading to unpredictable shifts in trophic dynamics (Soares et al. 2026). Nonetheless, essential oils derived from Negramina (*Siparuna guianensis*) and cloves have been shown to have a high selectivity against aphids without toxic effects on non-target beetles (Toledo et al. 2019; 2020).

The shift toward botanical inputs has also raised concerns regarding the abundance and diversity of soil microbiota (Soto-Barajas et al. 2025). High-concentration applications of antimicrobial EOs can cause transient suppressions of beneficial soil fungi and bacteria involved in nutrient cycling (Jouini et al. 2020; Leiva-Mora et al. 2025). Although BPs do not exhibit the multi-year persistence of synthetic legacy pesticides, the repetitive use of concentrated plant extracts can alter the soil microbiome’s composition, potentially affecting long-term agricultural fertility.

## Concluding remarks

In conclusion, halophyte plants represent a resilient and multifunctional resource for mitigating the compounding effects of climate change by offering sustainable alternatives to synthetic agrochemicals. Their rich reservoir of bioactive secondary metabolites, including terpenoids and phenolics, provides potent, multi-target biocidal activity against evolving agricultural pests and invasive weeds. Beyond pest control, halophytic biostimulants and their specialized root-associated microbiomes enhance crop productivity under salinity and drought stress by improving nutrient acquisition and upregulating antioxidant defense systems. While these botanical inputs reduce reliance on synthetic agrochemicals and their associated greenhouse gas emissions, translating promising laboratory and greenhouse findings into reliable field performance remains a critical challenge. Environmental factors such as UV radiation, volatilization, rainfall, temperature fluctuations, and soil interactions can reduce the stability, persistence, and efficacy of halophyte-derived bioactive compounds under field conditions while simultaneously altering exposure patterns for non-target organisms. Therefore, future research should prioritize robust multi-location field validation, the molecular characterization of complex herbicidal pathways, the optimization of nano-encapsulation and other advanced formulation technologies to enhance stability and controlled release, scalable production and application strategies, and long-term monitoring of ecotoxicological impacts on pollinators, beneficial soil microbiota, and other non-target organisms. Addressing these challenges will be essential to bridge the gap between experimental efficacy and practical agricultural implementation. Ultimately, the strategic domestication and integration of halophyte-based technologies have the potential to strengthen climate-resilient agriculture, safeguard ecosystem health, and help secure global food production amid rising salinity and increasing climatic uncertainty.

## Data Availability

Data sharing does not apply to this article as no datasets were generated or analyzed during the current study.
